# Effect of Cholecalciferol Supplementation on Inflammation and Cellular Alloimmunity in Hemodialysis Patients: Data from a Randomized Controlled Pilot Trial

**DOI:** 10.1371/journal.pone.0109998

**Published:** 2014-10-08

**Authors:** Lily Li, Marvin Lin, Maria Krassilnikova, Katya Ostrow, Amanda Bader, Brian Radbill, Jaime Uribarri, Joji Tokita, Staci Leisman, Vijay Lapsia, Randy A. Albrecht, Adolfo García-Sastre, Andrea D. Branch, Peter S. Heeger, Anita Mehrotra

**Affiliations:** 1 Division of Nephrology, Icahn School of Medicine at Mount Sinai, New York, New York, United States of America; 2 Department of Medicine, Icahn School of Medicine at Mount Sinai, New York, New York, United States of America; 3 Recanati Miller Transplantation Institute, Icahn School of Medicine at Mount Sinai, New York, New York, United States of America; 4 Immunology Institute, Icahn School of Medicine at Mount Sinai, New York, New York, United States of America; 5 Department of Microbiology, Icahn School of Medicine at Mount Sinai, New York, New York, United States of America; 6 Global Health and Emerging Pathogens Institute, Icahn School of Medicine at Mount Sinai, New York, New York, United States of America; 7 Division of Liver Diseases, Icahn School of Medicine at Mount Sinai, New York, New York, United States of America; Medical University of Graz, Austria

## Abstract

**Background:**

Memory T-cells are mediators of transplant injury, and no therapy is known to prevent the development of cross-reactive memory alloimmunity. Activated vitamin D is immunomodulatory, and vitamin D deficiency, common in hemodialysis patients awaiting transplantation, is associated with a heightened alloimmune response. Thus, we tested the hypothesis that vitamin D_3_ supplementation would prevent alloreactive T-cell memory formation in vitamin D-deficient hemodialysis patients.

**Methods and Findings:**

We performed a 12-month single-center pilot randomized, controlled trial of 50,000 IU/week of cholecalciferol (D_3_) versus no supplementation in 96 hemodialysis patients with serum 25(OH)D<25 ng/mL, measuring effects on serum 25(OH)D and phenotypic and functional properties of T-cells. Participants were randomized 2∶1 to active treatment versus control. D_3_ supplementation increased serum 25(OH)D at 6 weeks (13.5 [11.2] ng/mL to 42.5 [18.5] ng/mL, p<0.001) and for the duration of the study. No episodes of sustained hypercalcemia occurred in either group. Results of IFNγ ELISPOT-based panel of reactive T-cell assays (PRT), quantifying alloreactive memory, demonstrated greater increases in the controls over 1 year compared to the treatment group (delta PRT in treatment 104.8+/−330.8 vs 252.9+/−431.3 in control), but these changes in PRT between groups did not reach statistical significance (p = 0.25).

**Conclusions:**

D_3_ supplements are safe, effective at treating vitamin D deficiency, and may prevent time-dependent increases in T-cell alloimmunity in hemodialysis patients, but their effects on alloimmunity need to be confirmed in larger studies. These findings support the routine supplementation of vitamin D-deficient transplant candidates on hemodialysis and highlight the need for large-scale prospective studies of vitamin D supplementation in transplant candidates and recipients.

**Trial Registration:**

Clinicaltrials.gov NCT01175798

## Introduction

Alloreactive memory T-cells are pathogenic mediators of transplant injury. They are among the first cells to injure vascularized allografts in animal models [1], prevent tolerance induction in mice [2], and are associated with inferior transplant outcomes both in non-human primates and in human kidney transplant recipients [3]–[6].

Memory T-cells can be induced by direct exposure to alloantigens through transplantation, pregnancy, or blood transfusion. Evidence also indicates that heterologous (cross-reactive) immune responses initiated by environmental pathogens and homeostatic proliferation can facilitate the induction and expansion of alloreactive memory T-cells [7], [8]. Interventions to reduce graft injury caused by T-cell memory are not yet available.

The biologically active form of vitamin D, 1,25-dihydroxyvitamin D [1,25(OH)_2_D] has immune-modulatory properties. T-cells and dendritic cells (DCs), express the vitamin D receptor (VDR) and the 1α-hydroxylase needed to convert 25-hydroxyvitamin D [25(OH)D] to 1,25(OH)_2_D [9]. 1,25(OH)_2_D can directly modulate the function of T-cells and DCs through a variety of mechanisms [10]–[13], and can enhance tolerance induction in mice [14]. Importantly, many of the immune effects of vitamin D require local, immune cell production of 1,25(OH)_2_D from its immediate precursor, 25(OH)D, which circulates in blood [9], [15], [16].

Vitamin D deficiency is widespread among the general population [17], and is prevalent in patients with end-stage renal disease (ESRD) on hemodialysis (HD). The concentration of 25(OH)D is the main clinical indicator of vitamin D status and is used to define vitamin D deficiency. According to the Institute of Medicine (IOM), there is an elevation in the risk of experiencing adverse health consequences of vitamin D deficiency at 25(OH)D levels <20 ng/mL [18]. Over 80% of HD patients have serum 25(OH)D concentrations within the insufficient/deficient range despite ongoing treatment with activated vitamin D [1,25(OH)_2_D] for prevention of uremic bone disease [19], [20]. While there have been uncontrolled studies of nutritional vitamin D (D_2_ or D_3_) supplementation in HD patients and one short-term (3 week) randomized, controlled trial of D_3_ supplementation [20]–[23], the safety, efficacy, and *immunological* impact of nutritional vitamin D repletion in HD patients, particularly in patients already receiving 1,25(OH)_2_D, has not been tested in controlled studies.

Observational studies of kidney transplant recipients show an association between 25(OH)D deficiency and inferior graft outcomes [24], [25]. Our previously published data show a strong correlation between 25(OH)D deficiency and elevated memory T-cell alloimmunity in dialysis patients, many of whom are on the transplant waiting list, independent of known sensitizing events [19]. We hypothesized that inadequate concentrations of 25(OH)D contribute to the development of alloreactive memory T-cells by reducing the local synthesis of the 1,25(OH)_2_D that is needed to achieve optimal levels in immune cells. To test this hypothesis and to obtain preliminary data for power calculations for a larger multi-center study, we performed a pilot, single-center, randomized, controlled trial (RCT) of oral cholecalciferol (D_3_) (versus no supplementation) to assess the impact of increased 25(OH)D on memory T-cell alloimmunity, in patients already receiving 1,25(OH)_2_D as clinically indicated. Our data indicate that D_3_ supplementation is safe and effective at correcting vitamin D deficiency in this setting, and may prevent the time-dependent increase of peripheral cellular alloimmunity that occurs in many dialysis patients, establishing a strong rationale for conducting a larger, multi-center RCT to confirm these findings.

## Methods

### Clinical Trial Design

The protocol for this trial, sample case report form, and supporting CONSORT checklist are available as supporting information; see Protocol S1, Sample Case Report Form S1, and CONSORT Checklist S1. We recruited adults (≥18 years) on hemodialysis for ≥2 months at the Icahn School of Medicine at Mount Sinai (ISMMS) and Terrence Cardinal Cooke outpatient dialysis units in New York City from August 2010 to October 2011, excluding patients with a history of acute renal failure with potential for recovery, a history of HIV/AIDS, or inability to provide informed consent. All participants gave written informed consent, and the study was conducted according to the principles expressed in the Declaration of Helsinki. The study was approved by the Icahn School of Medicine at Mount Sinai (ISMMS) Institutional Review Board (IRB #09-2275) and registered at clinicaltrials.gov (NCT01175798).

Peripheral blood was obtained during the hemodialysis procedure. All study subjects were being dialyzed using biocompatible dialysis membranes. Serum 25(OH)D concentrations were determined commercially in a CLIA-certified laboratory (Nationwide Laboratories, Ft. Lauderdale, FL) by the chemiluminescent Diasorin method (www.diasorin.com, Stillwater, MN). Subjects with serum 25(OH)D<25 ng/mL were eligible for randomization. This cut-off for randomization was pre-determined based on previous data showing the distribution of vitamin D concentrations in the population [19].

The study was an open-label pilot RCT of nutritional Vitamin D- therapy (D_3_: cholecalciferol, purchased from BioTech Pharmacal, Inc: http://www.biotechpharmacal.com/). Participants were randomized in a 2∶1 ratio using Random Allocation Software (Version 1.0.0) [26] into one of two groups: oral cholecalciferol or no treatment (currently the standard of care at the dialysis units, as routine screening for and treatment of 25(OH)D deficiency are not part of practice guidelines for this population). Randomization was stratified by baseline 25(OH)D (10–24 ng/mL and <10 ng/mL). Within each stratum, randomization occurred in permuted 12-subject blocks. Implementation of randomization was performed by a study investigator.

Those participants randomized to D_3_ had a target 25(OH)D concentration of >35 ng/ml. The initial dose of D_3_ was 50,000/week for 6 weeks, at which time 25(OH)D was re-measured. Subjects whose 25(OH)D remained ≤35 ng/ml continued on 50,000 IU weekly for another 6 weeks, at which point 25(OH)D concentrations were repeated. Those subjects who achieved 25(OH)D concentrations >35 ng/ml were transitioned to 10,000 IU/week. D_3_ therapy was directly observed by a study coordinator or investigator. 25(OH)D was measured at baseline, 6 weeks, 3 months, and 6 months following randomization in both treatment and control groups, and other clinical information (routine labs, medication dosage) was collected at the same time-points.

The primary outcome was change in 25(OH)D at 6 weeks, 3 months, and 6 months. Secondary outcomes included change in clinical laboratory values (intact parathyroid hormone [PTH], serum calcium, and phosphorus), activated vitamin D requirements (mcg/treatment), and laboratory-based phenotypic and functional T-cell assays.

**Figure 1:**
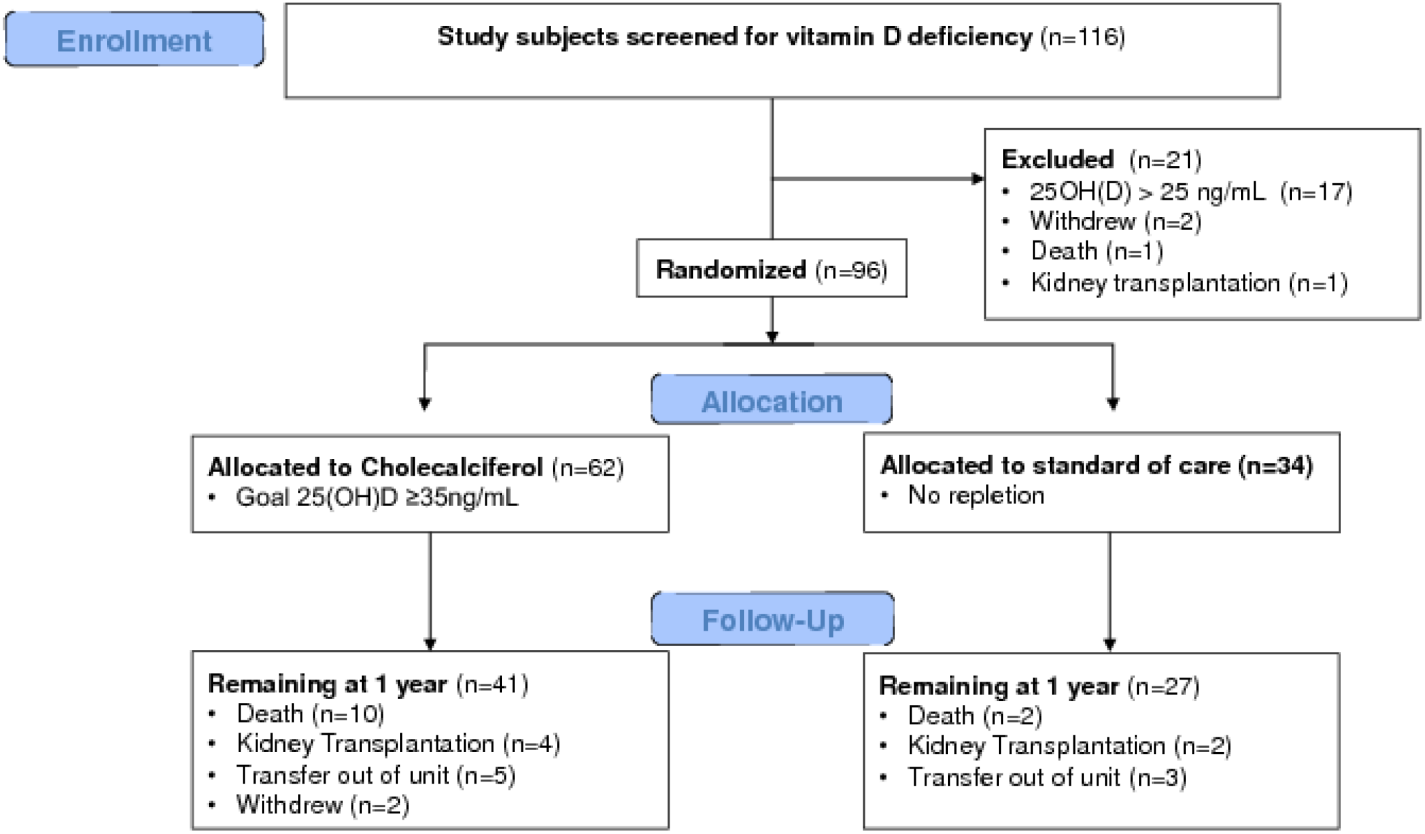
Study flow diagram. A total of 116 hemodialysis patients were screened, and 96 were randomized in a 2∶1 ratio to receive oral cholecalciferol (n = 62) or no repletion (n = 34). There were no differences in dropout rates from transplantation (6.6% vs 5.9% at 1 year, p = .90) or death (16.4% vs 5.9% at 1 year, p = .14) between groups. A total of 68 subjects completed follow-up to 1 year. Of those 68 subjects, 51 (34 in the treatment group and 17 in the control group) had sufficient PBMC samples meeting predetermined quality-assurance criteria for immunologic assessment.

All study subjects continued to receive routine care at their hemodialysis unit including monthly laboratory testing of calcium, phosphorus, and intact parathyroid hormone. Dosing of hemodialysis-related intravenous medications (activated vitamin D analog) continued as per pre-specified, standardized protocols in the hemodialysis units. Nursing staff adjusting hemodialysis-related intravenous medications were blinded to treatment assignment. Study subjects undergoing kidney transplantation during the study period were not included in the final analysis (Figure 1).

### Laboratory Procedures

Peripheral blood was collected during the hemodialysis procedure into BD Vacutainer tubes (BD, Franklin Lakes, NJ) containing sodium heparin for plasma or no additive for serum. PBMCs were isolated by Ficoll gradient centrifugation, cryopreserved at a concentration of 10^7^ cells/mL, and placed in a −80°C freezer for ≥24 hours prior to transfer to liquid nitrogen for long-term storage for ELISPOT assays. Serum separator tubes were left for one hour at room temperature before centrifugation at 1500 rpm for 15 minutes at 20°C, and serum was immediately divided into 0.5 mL aliquots and stored at −80°C until analysis.

10^6^ PBMCs were treated overnight with Streck Cell Preservative (Streck, Omaha, Nebraska) and stained with fluorochrome-labeled antibodies the following day as per the manufacturers’ recommendations for each antibody. Antibodies used for differentiation of T-cell subsets included: anti-CD4-FITC, anti-CD25-APC, anti-CD8-PE, anti-CD45RA-APC, anti-CD45RO-FITC (BD, Franklin Lakes, NJ), anti-CD4-PE-Cy7 and anti-Foxp3-PE (eBioscience, San Diego, CA). Stained cells were analyzed on a FACS Canto II flow cytometry (BD Bioscience, San Jose, CA, see Foxp3stainingeBioscience S1) and comparative results processed using FlowJo software (Tree Star, Inc, Ashland, OR).

IFNγ ELISPOT PRT assays were performed as previously described [5], [19], [27]. A total of 3×10^5^ live responder cells were incubated with 1×10^5^ of each allogeneic B cell line in duplicate or triplicate for 24 hours. Phytohemagglutinin (0.02 mg/mL) was used as a positive control. ELISPOT assays were developed according to standard operating protocol and spots quantified using an automated Immunospot Reader and CTL Immunospot Software (Cellular Technology Limited, Shaker Heights, OH). Mean numbers of IFNγ ELISPOTS from control wells (responder cells plus media only, typically <10 spots per well) were subtracted from the total number of spots in stimulated wells. Results are reported as the sum of the mean responses to each set of six stimulators [19].

Plasma anti-HLA antibody was assessed using HLA class I and class II antigen-coated microbeads (LABScreen Mixed, One Lambda Inc., Canoga Park, CA) and a flow analyzer (BioPlex 200 System with HTF, Bio-Rad, Hercules, CA) as previously described [28]. Determination of cut-off for reactivity was based on calculations as per the manufacturer’s software. A new reactivity was defined by an MFI <1000 at baseline that increased to ≥5000 at one year. Results were analyzed using HLA fusion software (One Lambda Inc., Canoga Park, CA).

### Statistical Analysis

Although the primary outcome was change in 25(OH)D concentrations, we attempted to power the study to detect differences in PRT results within groups over time, based on a standard deviation of 375 spots from our previous work [19], so as to provide estimates for power calculations for a larger, multi-center study. 30 subjects per group would provide 80% power to detect a difference of 200 spots in the PRT assay over time (alpha = 0.05). 30 study subjects per group would also provide 80% power to detect a difference in 25(OH)D levels *between* groups of 11 ng/mL at the 1 year time-point, based on a conservative estimate of the standard deviation of 15 ng/mL. Because we were unsure of how patients would respond to D_3_ supplementation, randomization was performed in a 2∶1 ratio to ensure an adequate number of patients had an increase in 25(OH)D levels over time, leading us to target an enrollment of 60 patients in the intervention group and 30 patients in the control group.

Statistical analysis was performed using SPSS version 20.0 (Chicago, IL) and GraphPad Prism 5 (GraphPad Software, Inc., La Jolla, CA). All analysis was performed on a per-protocol basis. There was no group cross-over, but, only patients reaching the 1-year time point were included in the 1-year data analysis in order to minimize the effect of missing data from patients lost to follow-up in the interim. Data are reported as medians and interquartile ranges or means and standard deviations for continuous variables, as appropriate, and as percentages of total for categorical variables. Baseline characteristics of the groups were compared using parametric (student’s t-test) or non-parametric (Mann-Whitney) tests for continuous variables, as appropriate, and Chi-square or Fisher’s exact tests for categorical variables, as appropriate. Changes over time were compared between groups using parametric (t-test) or non-parametric (Wilcoxon signed-rank test) tests, as appropriate. A two-tailed p-value<0.05 was considered statistically significant.

## Results

### Oral D_3_ supplementation is safe and effective

**Figure 2:**
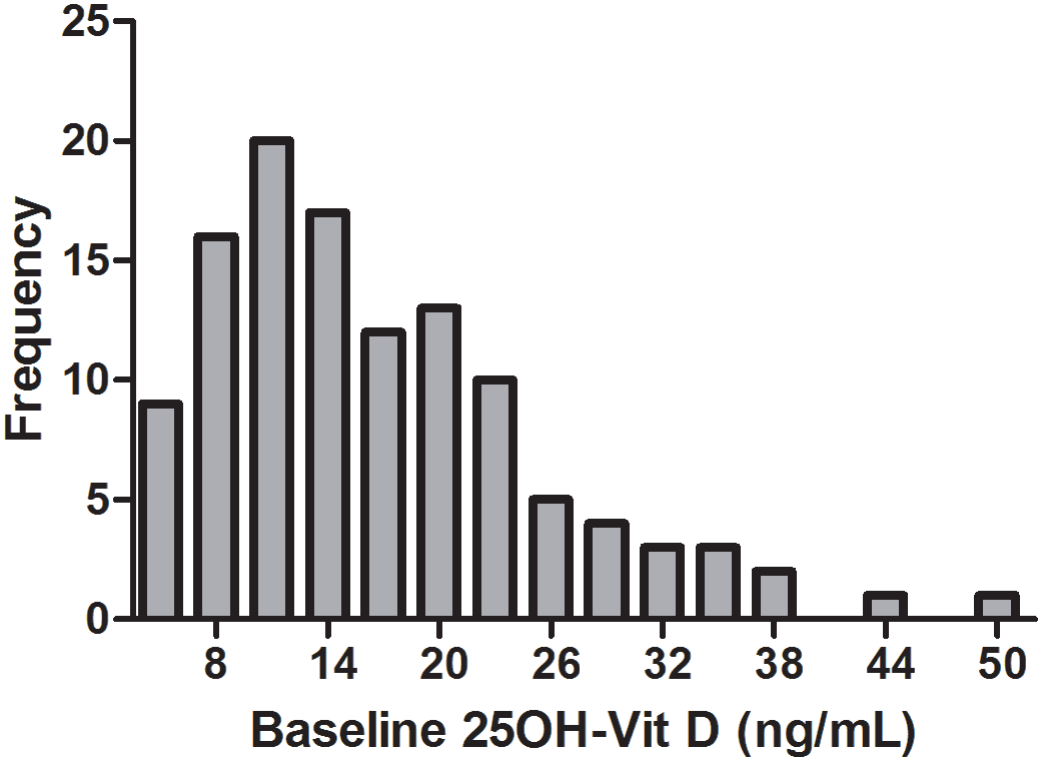
Baseline serum 25(OH)D concentrations in the study cohort. Distribution of serum 25(OH)D in the entire study cohort (n = 116) is shown. Median (IQR) 25(OH)D was 14.7 (10.5–21.6) ng/mL. 17 subjects with a baseline vitamin D level >25 ng/mL were excluded from randomization.

Of 116 chronic hemodialysis patients screened, 99 (85.3%) had 25(OH)D concentrations <25 ng/mL and qualified for randomization (**Figure 1**). The median (IQR) 25(OH)D concentration of the 116 patients (**Figure 2**) was 14.7 (10.5–21.6) ng/mL. 25(OH)D concentration was independent of month (time of year) of testing (data not shown). Reflecting the 2∶1 enrollment design, 62/99 (62.6%) were randomized to treatment with cholecalciferol (D_3_), and 34/99 (34.3%) were randomized to standard of care (control, no treatment); two patients withdrew, and one patient died prior to randomization (**Figure 1**). As randomization was stratified by baseline 25(OH)D concentration (10–24 ng/mL and<10 ng/mL), there were similar numbers of study subjects with baseline 25(OH)D<10 ng/mL in both groups (27.4% in the D_3_ group and 23.5% in the control group, p = 0.68). Demographics of the randomized patients (**Table 1**) showed no differences between treatment and control groups in baseline 25(OH)D, age, gender, race, dialysis vintage, history of diabetes, smoking status, and dialysis access (catheter vs. arteriovenous fistula/arteriovenous graft). The majority (61.5%) of randomized patients were of self-reported African ancestry.

**Table 1:** Baseline patient characteristics.

Characteristic	Treatment (n = 62)	Control (n = 34)	p-value
Baseline 25(OH)D, ng/mL (median, [IQR])	13.4 [9.3, 19.7]	13.1 [9.9, 18.6]	0.97
Age, years	60.2 [14.3]	58.9 [14.9]	0.66
Gender: Female	26 (41.9)	14 (41.2)	0.94
Race: Black	42 (67.7)	17 (50)	0.09
Dialysis Vintage, years (median, [IQR])	4.0 [1.3,8.0]	3.5 [1.2,7.9]	0.86
Diabetes	26 (41.9)	15 (44.1)	0.84
Current or Former Smoker	25 (40.3)	15 (44.1)	0.71
Dialysis Access: Catheter	12 (19.4)	8 (23.5)	0.45
Prior transplant	13 (21.0)	7 (20.6)	0.97
Prior blood transfusion	30 (48.4)	21 (61.8)	0.14
Prior pregnancy	20 (32.3)	13 (38.2)	0.31

Mean [SD] or count (%) unless otherwise specified.

**Figure 3:**
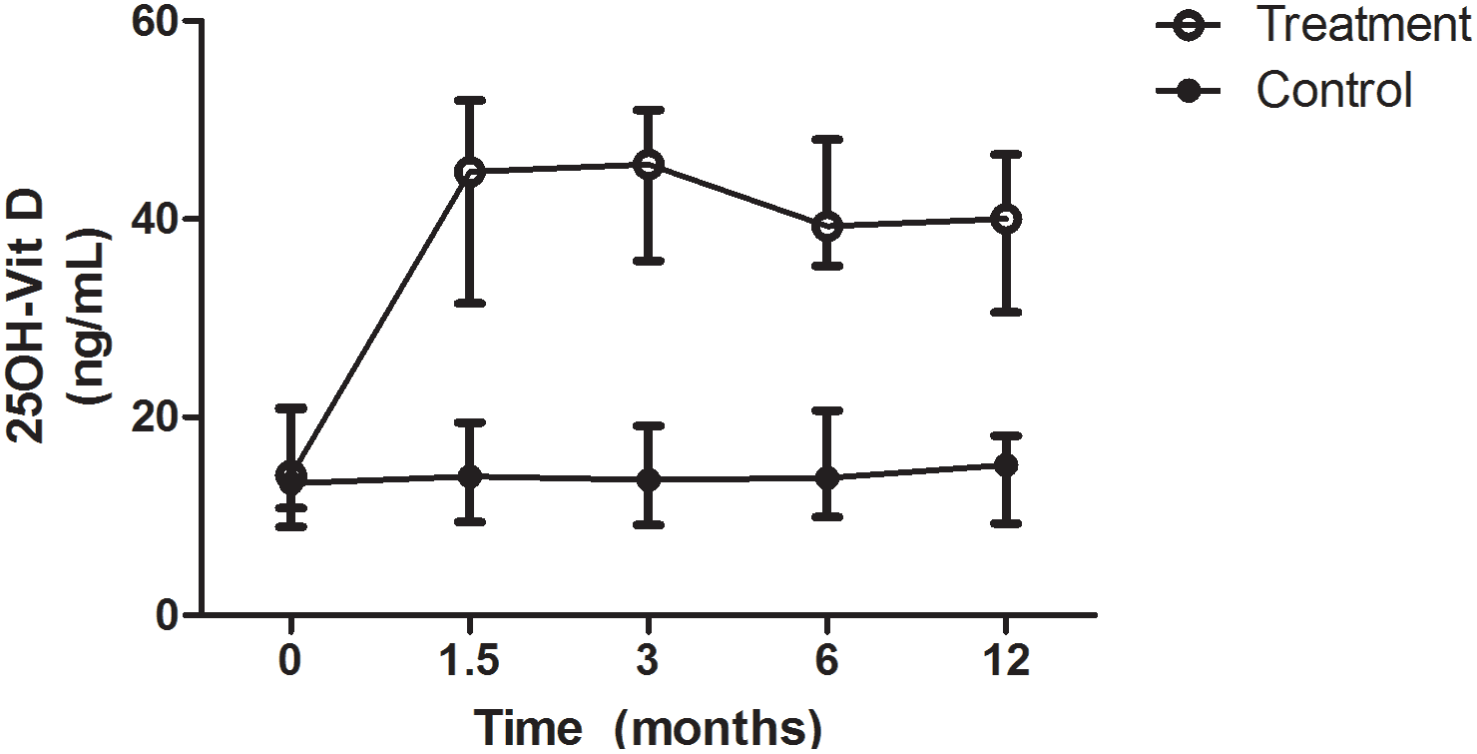
Effects of Vitamin D supplementation on serum 25(OH)D concentrations. Values increased by six weeks in the treatment group (13.5 [11.2] ng/mL to 42.5 [18.5] ng/mL, p<0.001), but remained low in the control group throughout the study duration. Median (IQR) are displayed on the graph.

Therapy with oral D_3_ resulted in a significant rise in serum 25(OH)D by 6 weeks (**Figure 3**), which was sustained throughout the 12 months of study. Serum 25(OH)D in the control group did not change (**Figure 3**). We did not observe any adverse events attributable to D_3_ supplementation (see AdverseEventsTable S1). There were no differences in dropout rates from transplantation (6.6% vs 5.9% at 1 year, p = .90) or death (16.4% vs 5.9% at 1 year, p = .14) between groups (**Figure 1**), and importantly, the overall mortality of 11.6% at 1 year was not higher than expected for the general dialysis population based on previous reports (approximately 20%) [29]. Serum calcium, phosphorus, intact PTH, and activated vitamin D requirements (paricalcitol) were similar in the control and intervention arms through the trial (**Table 2**), and no episodes of sustained hypercalcemia occurred in either group during the study (**Table 2**, see RawData S1).

**Table 2:** Laboratory parameters of bone and mineral metabolism and activated Vitamin D requirements.

	Treatment		Control		P-value*
	Baseline	12 Months	Baseline	12 Months	
25OH-D,** ng/mL (median, [IQR])	13.5 [9.8, 20.9]	40.9 [32.2, 45.5]	13.0 [10.9, 20.4]	15.8 [9.5, 19.5]	<0.001
PTH, pg/mL	465.0 (360.5)	555.9 (436.6)	434.3 (380.7)	637.4 (645.5)	0.35
Calcium, mg/dL	9.2 (0.6)	9.0 (0.7)	8.8 (1.0)	9.0 (1.0)	0.09
Phosphorus, mg/dL	5.8 (1.2)	5.3 (1.4)	5.4 (1.8)	5.1 (1.4)	0.95
Activated Vitamin D dose, mcg/treatment (median, [IQR])	4.0 [0, 9.1]	4.0 [2.5, 5.0]	3.5 [0, 5.6]	5.0 [2.3, 7.5]	0.22

Mean (SD) unless otherwise specified.

*p-values for comparison of change from baseline to 12 months between groups (treatment vs control).

**n = 41 in treatment group and 27 in control group.

### Effect of D_3_ supplementation on T-cell phenotype

**Figure 4:**
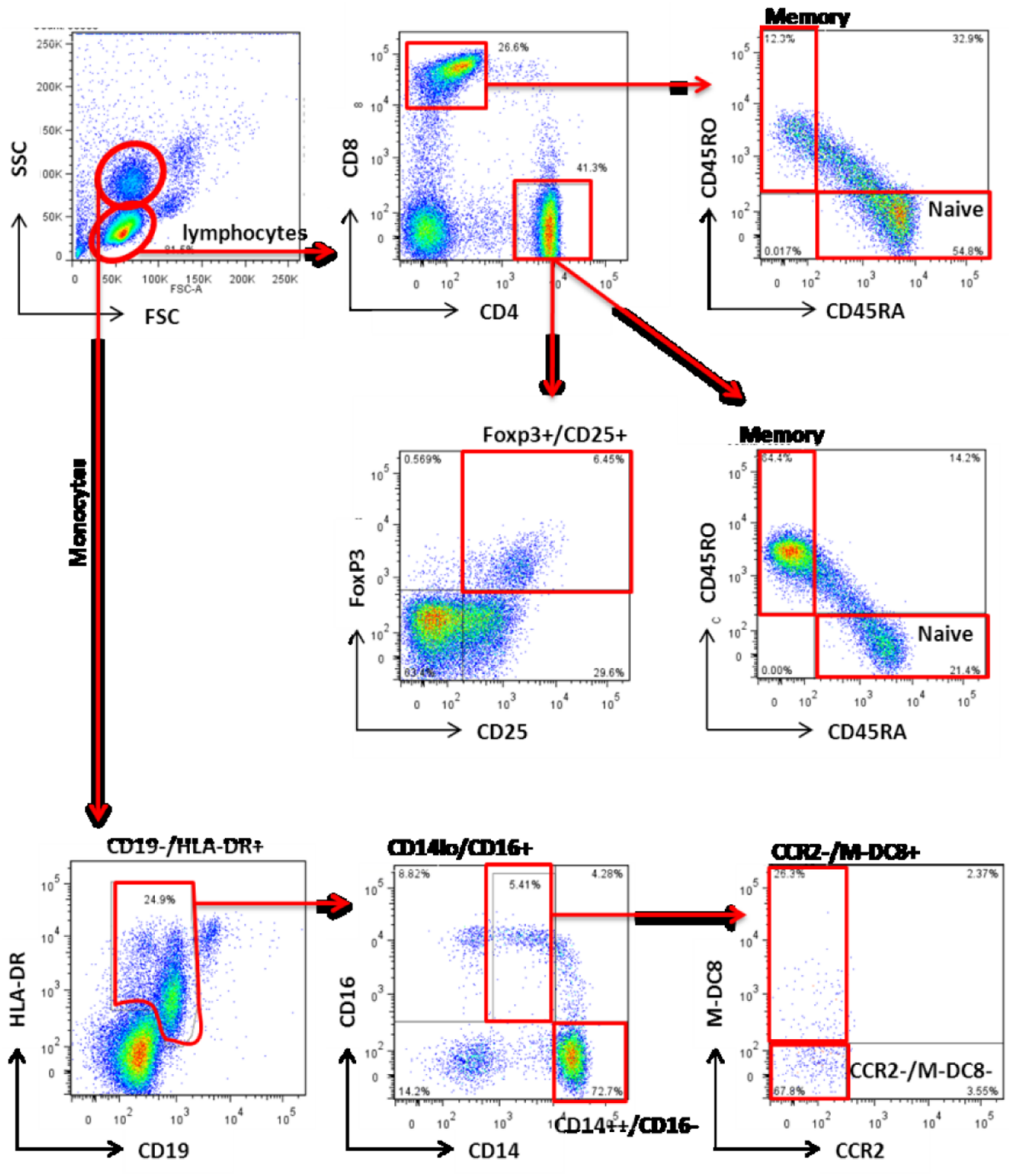
Gating strategy for enumeration of T cell and monocyte subsets. CD4 and CD8 memory (CD45RO+/CD45RA^neg^) and naïve (CD45RO^neg^/CD45RA+) T cells, and the Foxp3+/CD25+/CD4+ population containing regulatory cells were evaluated. Monocytes were identified by a CD19^neg^/HLA-DR+ phenotype, and further characterized into the CD14++/CD16^neg^ classical subset, and the CD14^lo^/CD16+ non-classical M-DC8+ and M-DC8^neg^ subsets.

We used flow cytometry to analyze and quantify PBMCs collected at baseline and 1 year. Gating strategies are shown in **Figure 4** and the data are summarized in **Table 3** **and** **Table 4**. Of 96 subjects randomized, 51 patients (34 in the treatment group and 17 in the control group) were available for 12 month follow-up, had sufficient PBMC, and were included in this analysis. None of the study subjects included in this analysis received packed red blood cell (PRBC) transfusions during the study period. At baseline, the median 25(OH)D concentration for these 51 patients was 14.1 ng/mL (IQR: 10.9–21.0 ng/mL), a value that did not differ from the parent cohort (14.7 ng/mL, IQR: 10.5–21.6 ng/mL). The baseline demographics of these subjects did not differ from those of the parent cohort (p>0.05 for each), supporting random and equal loss from the treatment and control groups, without evidence of bias.

**Table 3:** Effects of Vitamin D supplementation on T cell phenotypes†.

	Oral D_3_ (n = 34)		Control (n = 17)	
	Baseline	1 Year	Baseline	1 Year
**CD4%**	37.0 [29.0,45.4]	40.6 [29.9,47.1]	34.5 [22.3,39.5]	35.7 [27.8,40.6]
Naïve CD45RA+	33.6 [20.4,44.5]	32.9 [16.8,44.8]	31.3 [19.4,49.7]	34.7 [17.3,50.1]
Memory CD45RO+	52.7 [41.0,64.9]	50.6 [39.0,66.3]	61.9 [39.9,70.4]	49.2 [41.4,72.5]
Foxp3+CD25+	7.9 [5.9,9.3]	5.9 [2.8,9.0]	7.8 [4.6,10.9]	3.9 [1.4,7.6]
**CD8%**	17.1 [12.8,25.8]	14.0 [9.8,22.9]	17.7 [11.6,22.8]	15.8 [13.0,18.6]
Naïve CD45RA+	64.5 [55.8,80.0]	67.7 [52.3,77.0]	68.8 [51.5,79.5]	65.8 [47.6,79.9]
Memory CD45RO+	14.6 [7.1,19.3]	14.2 [8.0,21.7]	14.6 [8.8,26.7]	20.0 [9.0,27.9]

Values are expressed as median [IQR].

† no statistically significant differences in baseline, 1 year, or change from baseline to 1 year between groups.

**Table 4:** Effects of Vitamin D supplementation on monocyte subsets†.

	Oral D_3_ (n = 34)		Control (n = 17)	
	Baseline	1 Year	Baseline	1 Year
Total % Monocytes (CD19−/HLA-DR+)	6.4 [3.7,11.8]	6.2 [3.6,8.6]	5.5 [3.1,9.7]	5.9 [5.5,8.7]
Classical CD14++/CD16– % of monocytes	67.2 [53.4,78.5]	66.8 [57.2,78.2]	72.0 [64.2,79.5]	67.8 [52.5,73.4]
Nonclassical CD14lo/CD16+ % of monocytes CCR2−/M-DC8– CCR2−/MDC-8+	9.0 [4.8,16.4]2.5 [0.6,4.3] 1.8 [0.6,4.5]	10.6 [6.6,16.7]2.1 [1.0,3.6] 0.9 [0.5,1.8]	9.5 [6.4,13.8]1.5 [0.6,2.9] 1.2 [0.5,3.3]	10.3 [5.3,16.4]2.0 [1.7,5.1] 1.3 [0.6,2.9]

† no statistically significant differences in baseline, 1 year, or change from baseline to 1 year between groups.

We examined the percentages of CD4 and CD8 T-cells including their naïve (CD45RA+/CD45RO^neg^) and memory (CD45RA^neg^/CD45RO+) subsets and quantified CD4+Foxp3+ T-cells. These analyses showed no differences in memory or naïve CD4 or CD8 cell subsets between the groups at baseline and at 1 year (**Table 3**). The percentages of CD4+Foxp3+ T-cells were lower in both groups at 1 year versus baseline, but the values did not differ between groups at either time point (**Table 3**).

### D_3_ supplementation may prevent time-dependent increases in cellular alloimmunity

We used an IFNγ ELISPOT-based panel of reactive T-cell (PRT) assay to quantify the frequencies of alloreactive memory T-cells in each patient. In this assay, PBMC are stimulated with a panel of HLA-typed allogeneic B cell lines inducing alloreactive memory, but not naïve, T-cells to produce IFNγ [3]. Our previously published work showed that the summed responses to a randomly selected panel of 6 primary B cell lines is sufficient to provide reproducible and interpretable information [19], and that subjects with the highest PRT results were at an elevated risk for developing post-transplant acute rejection and inferior one year kidney function [5].

**Figure 5:**
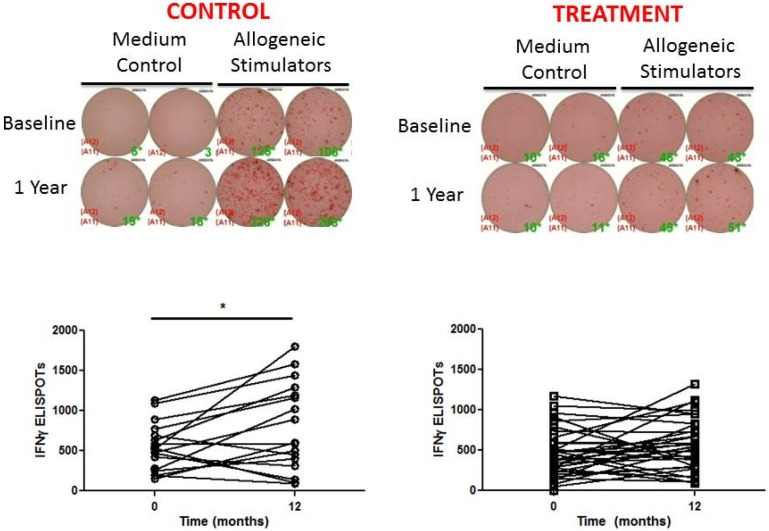
Vitamin D supplementation may prevent the time-dependent increase in PRT. (A) Representative ELISPOT PRT wells in duplicate at baseline and one year with no stimulation (media control) or response to allogeneic B cells. (B) Quantified results reveal a significant increase in the number of IFNγ ELISPOTs over time in the control group (517.4+/−280.8 to 797.8+/−542.3 spots, p = 0.03), but the comparison of “delta” PRT (1 year – baseline) in the treatment vs control group did not reach statistical significance (104.8+/−330.8 in treatment vs 252.9+/−431.3 in control, p = 0.25).

**Figure 6:**
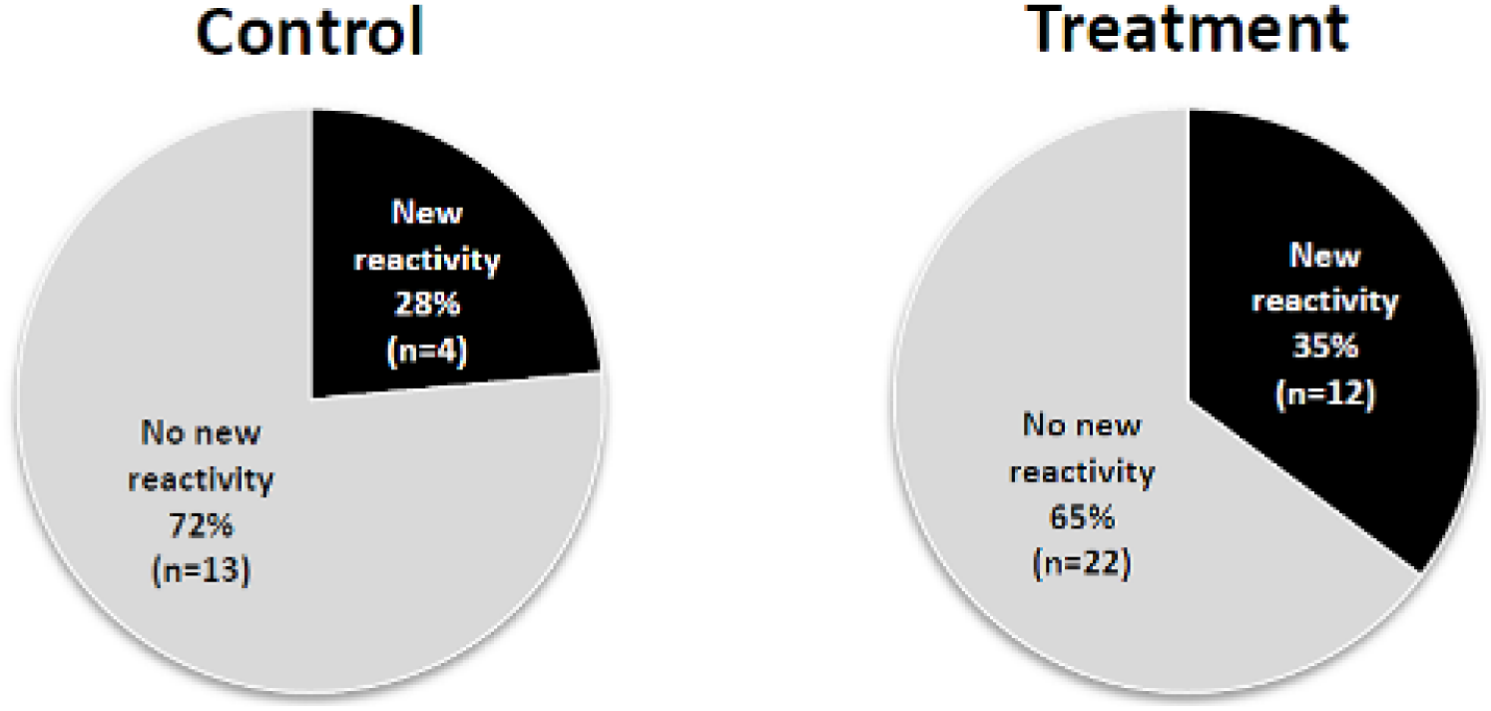
Vitamin D supplementation does not alter anti-HLA antibodies. Pie charts depicting the percentages of patients in each group that developed new anti-HLA antibodies between entry and 1 year (p = 0.393). A new reactivity was defined as having a MFI <1000 at baseline and >5000 at one year using the LuminexPRA assay.

The PRT results were similar in the active treatment and control groups at baseline (450.9+/−301.2 vs 517.4+/−280.8 spots, p = 0.41) (**Figure 5**). Whereas the PRT did not change over 1 year study in the patients treated with oral D_3_, the PRT increased significantly in the controls (517.4+/−280.8 to 797.8+/−542.3 spots, p = 0.03) (**Figure 5**). However, when comparing the “delta” PRT (1 year – baseline) in the treatment vs control group, this comparison did not reach statistical significance (delta PRT in treatment 104.8+/−330.8 vs 252.9+/−431.3 in control, p = 0.25). D_3_ supplementation did not alter the development of new alloantibodies (**Figure 6**).

### Effect of D_3_ supplementation on peripheral pro-inflammatory monocytes

Peripheral blood monocytes are a heterogenous population of mononuclear cells capable of modulating inflammation and activating T cells, regulating expression of MHC, co-stimulatory molecules, and cytokines. Previous work by others showed that “non-classical” CD14^lo^/CD16+ monocytes produce higher amounts of pro-inflammatory cytokines and express more cell surface HLA-DR than their classical CD14++/CD16^neg^ monocyte counterparts [30]. We did not observe differences in either of these monocyte subsets, either between groups at baseline and 1 year, or between groups over time (**Table 4**).

The CD14^lo^/CD16+ non-classical monocyte population is comprised of two subgroups: M-DC8+ and M-DC8^neg^ cells. The M-DC8+ subset has been shown to express two-fold more HLA class I and II molecules, to produce higher amounts of IL-12, and to induce more potent Th1 immune responses for the priming of cord blood T cells versus the M-DC8- population [31]. While there appeared to be a time-dependent, 50% decrease in M-DC8+ monocytes in the treatment group (**Table 4**), this change over time was not statistically significantly different from changes in the control group over time.

## Discussion

In this pilot RCT of D_3_ supplementation in vitamin D-deficient hemodialysis (HD) patients, we demonstrate safety and efficacy of D_3_ in the setting of concurrent therapy with activated vitamin D analogs. Subjects randomized to D_3_ therapy did not experience sustained hypercalcemia or other adverse events. While our study was not powered to examine mortality, the overall mortality of the cohort did not exceed that of the dialysis population in general [29] and published data show a survival benefit from vitamin D supplementation in other populations [32].

We newly show that D_3_ supplementation may inhibit the expansion of alloreactive T-cell memory over 1 year on dialysis. Although the comparison of “delta” PRT (1 year – baseline) in the treatment vs control group did not reach statistical significance, (delta PRT in treatment group: 104.8+/−330.8 vs 252.9+/−431.3 in control group, p = 0.25), this pilot study provides important data on standard deviation and effect size that can be used to design and power a larger clinical trial. If confirmed in a larger RCT, this would be the first demonstration of a potentially clinically relevant effect of nutritional vitamin D supplementation (D_3_) that could impact standard of practice. These data expand upon our prior cross-sectional observations [19] and suggest that vitamin D supplements may prevent the induction of environmentally driven, heterologous (cross-reactive) cellular alloimmunity. The results support the therapeutic use of nutritional vitamin D (D_2_ or D_3_) in all hemodialysis patients, especially those on the transplant waiting list who may benefit from vitamin D’s inhibitory effects on alloimmunity. We demonstrated potential effects of D_3_ supplementation on cellular alloresponses in just one year; as most HD patients on the deceased-donor waiting list wait from 2–7 years for a transplant, and the risk for developing cross-reactive anti-donor T-cell immunity increases with longer HD vintage [33], the impact of vitamin D deficiency on heterologous immunity over time may be even greater, and needs to be evaluated in larger prospective multi-center longitudinal studies.

Our findings are consistent with emerging evidence in the literature that deficiency of 25(OH)D (despite exogenous 1,25(OH)_2_D therapy) reduces local immune cell production of active vitamin D, and thereby promotes inflammation [16], [34]. While hemodialysis patients are commonly treated with 1,25(OH)_2_D analogs for the prevention of renal osteodystrophy, there are currently no recommendations for the regular surveillance or repletion of the precursor, 25(OH)D, and the prevalence of vitamin D deficiency in this population is high [20]. Vitamin D has been reported to inhibit dendritic cell (DC) differentiation and maturation and impair DC antigen presentation to attenuate the alloreactive T-cell response [10], [11]. Consistent with these observations, we noted fewer proinflammatory monocytes in the treatment arm over time (**Table 4**), suggestive of an inhibitory effect on APCs, but the difference in this change between treatment and control groups was not statistically significant. Vitamin D has also been reported to promote Th2 immunity while limiting Th1 responses and to inhibit antigen-induced T-cell activation [12], [13], which could prevent induction of cross-reactive alloimmunity. We acknowledge that results from this limited RCT do not reveal the mechanisms that underlie our findings; however, they suggest that vitamin D supplementation impacts alloreactive memory formation through a process that is likely to involve local production of 1,25(OH)_2_D and provide a foundation for future detailed mechanistic studies by our group and others.

Observational studies in the kidney transplant population show an association between 25(OH)D deficiency and inferior graft outcomes [24], [25], [35]. In addition, a functional polymorphism in the vitamin D receptor (VDR) (*FokI*, rs10735810) leading to increased transcriptional activity has been shown to correlate with improved renal allograft survival [36]. Results of our immunological studies are consistent with these clinical observations, and highlight the need for prospective trials of vitamin D therapy in kidney transplant candidates and recipients with a focus on graft and patient outcomes, possibly stratifying by polymorphisms in the VDR and other genes involved in vitamin D metabolism and function.

Significant limitations of our study include the un-blinded nature and lack of a placebo control, thus introducing the possibility of study subject and investigator bias. However, all laboratory-based assays were performed by technicians unaware of study subject treatment assignment in an effort to minimize investigator bias. Because patients were aware of group assignment, we cannot exclude the possibility that some patients assigned to the control group obtained vitamin D supplements elsewhere. However, as 25(OH)D concentrations remained stable over time in the control group, it is unlikely that these patients were taking clinically significant doses of any vitamin D supplements.

In conclusion, the results of this pilot RCT show that nutritional vitamin D (D_3_) supplementation is safe and effective in vitamin D-deficient HD patients concurrently treated with 1,25(OH)_2_D, and may prevent time-dependent increases in memory T-cell alloreactivity. Because memory T-cell alloreactivity pre-transplantation negatively affects post-transplant outcomes, and because vitamin D deficiency is associated with higher rates of acute rejection in kidney transplant recipients [35], treatment with vitamin D has the potential to improve kidney transplant outcomes and needs to be evaluated prospectively in transplant candidates/recipients. Given the apparent safety and low cost of vitamin D supplements, routine screening and correction of 25(OH)D deficiency should be considered in all dialysis units, with monitoring and reporting of the risks and benefits observed in a larger population.

## Supporting Information

Adverse Events Table S1 **Tabular view of adverse events.**
(DOCX)

CONSORT Checklist S1 **Completed CONSORT checklist for randomized controlled trials referencing the manuscript.**
(DOC)

Foxp3stainingeBioscience S1 **Instructions for Foxp3 intracellular staining.**
(PDF)

Protocol S1 **IRB-approved study protocol detailing all study-related procedures.**
(DOC)

Raw Data S1 **Excel spreadsheet of raw data from the study.**
(XLSX)

Sample Case Report Form S1 **Sample case report form from the trial used for data collection.**
(DOCX)
