# Supplementary material for: Effect of Cholecalciferol Supplementation on Inflammation and Cellular Alloimmunity in Hemodialysis Patients: Data from a Randomized Controlled Pilot Trial
Source: PLoS One. 2014 Oct 8;9(10):e109998. doi: 10.1371/journal.pone.0109998 (PMC4190314; doi:10.1371/journal.pone.0109998)
Supplement: Adverse Events Table S1 — Tabular view of adverse events. (DOCX) [file pone.0109998.s001.docx]

**Adverse Events**

| Adverse Event* | **Treatment (D3) (n=62)** | **Control (n=34)** |
| --- | --- | --- |
| Death | 10 (16.4%) | 2 (5.9%) |
| Subjects with hypercalcemia (Ca > 10.5 mg/dL) at any point during the study | 7 (11.3%) | 2 (5.9%) |
| Subjects with sustained hypercalcemia (defined as Ca > 10.5 mg/dL for > 4 weeks) | 0 (0%) | 0 (0%) |
| Withdrawal from the study at subject request | 2 (3.2%) | 0 (0%) |

*none of the comparisons above are statistically significant
