# Supplementary material for: Effect of Cholecalciferol Supplementation on Inflammation and Cellular Alloimmunity in Hemodialysis Patients: Data from a Randomized Controlled Pilot Trial
Source: PLoS One. 2014 Oct 8;9(10):e109998. doi: 10.1371/journal.pone.0109998 (PMC4190314; doi:10.1371/journal.pone.0109998)
Supplement: Foxp3stainingeBioscience S1 — Instructions for Foxp3 intracellular staining. (PDF) [file pone.0109998.s003.pdf]

Foxp3 PE

Catalog Number(s): 9012-4776-025 (25 tests), 12-4776-120 (120 tests)

CE IVD

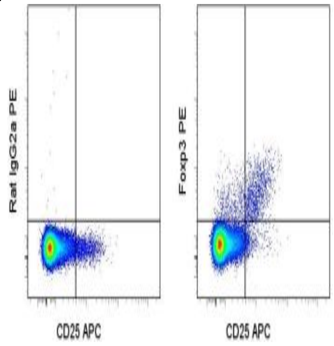

Staining of normal human peripheral blood lymphocytes with CD25 APC followed by intracellular staining with Rat IgG2a PE (left) or Foxp3 PE (right).

Product Information

Contents: Foxp3 PE

REF Σ **Catalog Number(s):** 9012-4776-025 (25 tests), 12-4776-120 (120 tests)

Clone: PCH101

Concentration: 5 uL (0.25 ug)/test (a test is defined as the amount that will stain 1 x 10e6 cells in 100 uL)

Host/Isotype: Rat IgG2a, kappa

Formulation: Aqueous buffer, 0.09% sodium azide, may contain carrier protein/stabilizer.

Storage Conditions: Store at 2-8°C. Do not freeze.

Light-sensitive material.

Caution, contains Azide

Manufacturer: eBioscience, Inc., 10255 Science Center Drive, San Diego, CA 92121, USA

EC REP **Authorized Representative:** Bender MedSystems GmbH, an eBioscience Company Campus Vienna Biocenter 2 A-1030 Vienna Austria

Intended Use

The PCH101 fluorochrome-conjugated monoclonal antibody reacts with the human FoxP3antigen. FoxP3 can be detected in human biological samples using immunological techniques.

Principles of the Test

Flow cytometry is a useful tool for simultaneously measuring multiple physical properties of individual particles (such as cells). Cells pass single-file through a laser beam. As each cell passes through the laser beam, the cytometer records how the cell or particle scatters incident laser light and emits fluorescence. Using this flow cytometric analysis protocol, one can perform a simultaneous analysis of surface molecules at the single-cell level.

Description

The PCH101 antibody reacts intracellularly with the amino terminus of human Foxp3 protein also known as FORKHEAD BOX P3, SCURFIN, and JM2. Foxp3, a 49-55 kDa protein, is a member of the forkhead/winged-helix family of transcriptional regulators, and was identified as the gene defective in ‘scurfy’ (sf) mice. Constitutive high expression of Foxp3 mRNA has been shown in CD4+CD25+ regulatory T cells (Treg cells), and ectopic expression of Foxp3 in CD4+CD25- cells imparts a Treg phenotype in these cells.

Not for further distribution without written consent. Copyright © 2000-2010 eBioscience, Inc.

Specimen Collection and Storage Instructions

Collect venous blood sample by venipuncture into a sterile blood collection tube using an appropriate anticoagulant (EDTA is recommended). Keep samples at room temperature (18-25°C). Prior to use, mix samples by gentle agitation.

Materials Required But Not Provided

- 12x75 mm test tubes
- Buffers (eBioscience Flow Cytometry Staining Buffer, Cat. No. 00-4222 recommended)
- Lysis Buffer (eBioscience 1X RBC Lysis Buffer, Cat. No. 00-4333 or eBioscience 1-step Fix/Lyse Solution (10X), Cat. No. 00-5333 recommended)
- For intracellular staining use IC Fixation Buffer and Permeabilization Buffer, Cat. No. 88-8823 (intracellular cytokine or cytoplasmic protein staining) or Foxp3 Buffer Set, Cat. No. 00-5523 (For nuclear protein staining). Refer to the Best Protocols section of the eBioscience website for the "Staining Intracellular Antigens for Flow Cytometry" protocols.
- Viability stain (7-AAD Viability Staining Solution, Cat. No. 00-6993 or Propidium Iodide Staining Solution, Cat. No. 00-6990 recommended)
- Automated pipettes
- Centrifuge
- Vortex mixer
- Ice bucket or refrigerator
- Flow cytometer

Test Protocol

NOTE: For intracellular staining, refer to the Best Protocols section of the eBioscience website for the "Staining Intracellular Antigens for Flow Cytometry" protocols.

1. Aliquot 100 µL of the test sample into tubes.
2. Add 5 µL of the appropriate antibody to each tube.
3. Incubate 30-60 minutes at 2-8°C. Alternatively, samples can be incubated at room temperature in the dark 15-30 minutes.
4. Add 2 ml of 1X RBC Lysis Buffer (at room temperature) per tube. Mix gently. (Alternatively, samples can be incubated with 2 mL 1-step Fix/Lyse Solution.)
5. Incubate samples in the dark at room temperature for 10 minutes. Do not exceed 15 minutes of incubation with the RBC Lysis Buffer.
6. Centrifuge samples at 300-400 x g for 5 minutes at room temperature, decant/aspirate supernatant and wash 1 time with 2 ml of Flow Cytometry Staining Buffer.
7. Centrifuge samples at 300-400 x g for 5 minutes at room temperature, decant/aspirate supernatant.
8. Resuspend stained cell pellet in 1 mL Flow Cytometry Staining Buffer and analyze samples on a flow cytometer.

Limitations

1. For optimal performance of fluorochrome conjugated antibodies, store vials at 2-8°C in the dark. Do not freeze.
2. Centrifuge the antibody vial prior to opening to recover the maximum volume.
3. Except where noted in the protocol, all staining should be done on ice or at 2-8°C with minimal exposure to light.

Performance Characteristics

Consistency of high-quality reagents is ensured by testing each lot of monoclonal antibody for conformance against characteristics of a standard reagent. Representative flow cytometric data is included where appropriate.

Evidence of Deterioration

For questions or concerns regarding the performance or quality of products received, please contact eBioscience Technical Support (see below).

References

Procedures for the Collection of Diagnostic Blood Specimens by Venipuncture (H3-A6), 3rd Edition published by the National Committee for Clinical Laboratory Standards.

Brunkow ME, Jeffery EW, Hjerrild KA, et al. Disruption of a new forkhead/winged-helix protein, scurfin, results in the fatal lymphoproliferative disorder of the scurfy mouse. Nature Genetics. 2001; 27(1):68-73.

Wildin RS, Ramsdell F, Peake J, et al. X-linked neonatal diabetes mellitus, enteropathy and endocrinopathy syndrome is the human equivalent of mouse scurfy. Nature Genetics. 2001; 27(1):18-20.

Not for further distribution without written consent. Copyright © 2000-2010 eBioscience, Inc.
www.eBioscience.com ORDER INQUIRIES • +43 1 796 40 40-120 TECH • +43 1 796 40 40-0 europe@eBioscience.com
