# Supplementary material for: Effect of Cholecalciferol Supplementation on Inflammation and Cellular Alloimmunity in Hemodialysis Patients: Data from a Randomized Controlled Pilot Trial
Source: PLoS One. 2014 Oct 8;9(10):e109998. doi: 10.1371/journal.pone.0109998 (PMC4190314; doi:10.1371/journal.pone.0109998)
Supplement: Protocol S1 — IRB-approved study protocol detailing all study-related procedures. (DOC) [file pone.0109998.s004.doc]

**Immunologic Impact of Vitamin D Repletion in Hemodialysis Patients: A Randomized Controlled Trial**

PI: Peter Heeger MD

Mount Sinai School of Medicine

Contact: Anita Mehrotra MD

One Gustave L. Levy Place Box 1243

New York, NY 10029

(212)-241-8004

Anita.Mehrotra@mssm.edu

**TABLE OF CONTENTS**

ITEM PAGE NUMBER

Study Summary 3

Introduction 3-6

Background and Rationale 3-5

Study Aims 5

Hypotheses 5

Trial Timeline 6

Study Design and Procedures 7-8

Inclusion Criteria 7

Exclusion Criteria 7

Enrollment/Screening 7

Randomization 7-8

Follow-Up Visits 8

Study Modality 8

Dosage and Administration 8 Study Procedures 8

Planned Statistical Analysis 8

Safety Evaluations 9-10

Study Monitoring 11

Study Completion Procedures 11

References 11-14

WORKING RESESARCH PROTOCOL: Immunologic Impact of Vitamin D Repletion in Hemodialysis Patients: A Randomized Controlled Trial

Study Summary

Innate and adaptive immunity are commonly impaired in patients with end stage renal disease (ESRD) on dialysis.^1-11^ The myriad of immune defects in these patients, often attributed to uremia, may account for their high risk of bacterial infection ^12^ and suboptimal responses to vaccination. ^11^ The mechanisms underlying these abnormalities in immune function remain elusive, but emerging evidence indicates that 25OH-Vitamin D exerts potent and complex control over innate and adaptive immunity.^13-19^ Vitamin D deficiency is common in dialysis patients,^20^ and the immune effects associated with 25OH-Vit D deficiency overlap with those found in many dialysis patients. The kidney is the dominant site of 1-alpha-hydroxylase activity required for producing active 1,25OH-Vit D; however, immune cells also express the 1-alpha-hydroxylase enzyme.^21-29^ Evidence indicates the effects of Vitamin D on modulating immunity require conversion of 25OH-Vit D to1,25OH-Vit D within the immune cells (rather than via circulating 1,25OH-Vit D).^13^ As a consequence, total body deficiency of 25OH-Vit D can impact immune function despite ongoing therapy with active 1,25OH-Vit D (which most dialysis patients are receiving). Our preliminary data confirm the high prevalence of 25OH-Vit D deficiency in dialysis patients and show that Th1 T cell alloimmunity is stronger in patients deficient in 25OH-Vit D, supporting the hypothesis that Vit D deficiency has important immunological consequences. Based on the published literature and our preliminary data, we hypothesize that repletion of 25OH-Vit D enhances immunity in dialysis patients. To test this hypothesis, we propose a randomized controlled trial of oral 25OH-Vit D repletion in this patient population. One hundred fifty 25OH-Vit D deficient study subjects will be randomized to either treatment with 50,000 IU oral 25OH-Vit D weekly or no treatment (standard of care). The primary outcome of change in 25OH-Vit D level will be measured at 6 weeks, 3 months, 6 months, and 12 months. Secondary outcomes to be measured include change in peripheral blood mononuclear cell (PBMC) profile by flow cytometry at 6 and 12 months, change in ELISPOT-based panel of reactive T cell (PRT) readout at 6 and 12 months, change in PMBC cytokine production in response to toll-like-receptor stimulation at 6 and 12 months, and response to influenza vaccination.

INTRODUCTION

Background and Scientific Rationale

*Immune dysfunction in dialysis patients*: Patients on dialysis have a significantly higher rate of infection^12^ and sepsis ^30^ than the general population, have lower rates of developing protective immunity in response to vaccinations ^31-33^, and have higher rates of symptomatic tuberculosis (TB) infections ^34-36^, among other clinical parameters. While the mechanisms underlying these findings are not fully understood, experimental observations indicate a myriad of disturbances in function of both innate and adaptive immune systems in patients on dialysis. Among the abnormalities found in innate immunity are defective intracellular killing of bacteria ^37^, reduced macrophage synthesis of nitric oxide ^37^, alterations in TLR signaling pathways ^4,38^, and increased production of IL-6, TNFa, IL-1 and IL-12 by monocyte/macrophages.^5^ In terms of adaptive immunity, dialysis is associated with diminished T cell proliferative responses to mitogens and superantigens ^39-40^, along with decreased expression (and function) of MHC, costimulatory and adhesion molecules on antigen presenting cells (APCs).^39-43^ T cell responses are skewed toward Th1 (IL-12, IFNg) and away from IL-4, IL-10 immunity in dialysis patients.^44^ Other observations include a paradoxical increase in T cell activation markers (e.g. CD69).^45^ While etiologies are not known, the observed disturbances have been ascribed to the uremic state, the hemodialysis procedure, and complications of chronic kidney failure among other explanations.^1,3,46^ Still, large gaps remain in our understanding of immunity in this patient population.

*Vitamin D and bone metabolism*: Vitamin D_2_ and D_3_ are obtained from dietary sources and from conversion in the skin via UV light, and are 25-hydroxylated in the liver (via enzyme CYP2R1, among others).^14^ It is the 25OH-Vit D level that determines an individual’s total body Vitamin D “status.”^13^ In the context of normal renal function, 25OHVit- D undergoes 1α hydroxylation to its 1,25 active form by CYP27B1 in the kidney, an enzyme that is tightly regulated by PTH, serum calcium and phosphorus.^14^ Active 1,25OH-Vit D binds to its intracellular receptor (vitamin D receptor, VDR), increasing calcium and phosphorus uptake by the intestine and inhibiting PTH release. Catabolism occurs via CYP24-mediated 24-hydroxylation, an enzyme that is upregulated by 1,25OHVit D and functions as a negative feedback loop.

Because renal CYPR27B1 is essentially absent in patients with ESRD, levels of circulating 1,25OH-Vit D are commonly well below the normal range, contributing to secondary hyperparathyroidism and bone loss. As a result, dialysis patients with evidence of hyperparathyroidism and abnormalities in bone metabolism are treated with active 1,25OH-Vit D. Nonetheless, total body Vit D (25OH-Vit D) deficiency is common in dialysis patients; approximately 85% have been reported to have levels below the normal range.^20^ While current thinking suggests that treatment with active 1,25OH-Vit D is sufficient for suppression of PTH secretion and treating bone disease in dialysis patients, whether 25OH-Vit D deficiency has an independent effect on bone metabolism in dialysis patients is not known. One recent study of oral 25OH-Vit D repletion in dialysis patients indicated that 1) it normalized total body Vit D 2) enhanced ability to achieve Kidney Disease Outcomes and Quality Initiative (KDOQI) targets for Ca and Phos, 3) lowered requirements for 1,25OH-Vit D therapy and 4) tended to normalize bone alkaline phosphatase levels, all in the absence of toxicity.^20^ These findings supplemented by evidence of increased mortality ^47^ and immune deficiency in Vit D-deficient dialysis patients support the need for additional studies.

*Vitamin D and the immune system*: Work performed over the last decade has shown than many non-renal cells including immune cells (monocytes, macrophages, dendritic cells and T cells), express the VDR, respond to 1,25OH-Vit D, and possess enzymatic machinery to generate 1,25OH-Vit D from its circulating 25-OH precursor.^22-23,25-27,48-50^ The importance of these findings is that 1) 1,25OH-Vit D has potent immunomodulatory effects and 2) many of these immune functions are dependent upon local production of 1,25OH-Vit D by the immune cells rather than direct effects of circulating 1,25OH-Vit D produced by the kidney. The best understood example of how 25OH-Vit D impacts innate immunity relates to intracellular killing of bacteria, specifically Mycobacterium tuberculosis (MTb).^37^ Macrophages increase VDR and CYP27B1 (1-alpha-hydroxylase) expression following toll-like receptor (TLR) stimulation by MTb or LPS. In the context of normal serum 25OH-Vit D (>30ng/ml), this circulating precursor is taken up by the macrophage and is converted to its active 1,25 form by CYP27B1. The active Vit D binds to the VDR in the macrophage and is transported to the nucleus where it leads to upregulation of >200 gene products. Of note, the promoter regions for cathelicidin and defensin B2, 2 antimicrobial peptides, contain consensus VDR response elements that bind to the VDR/1,25 complexes.^51^ 1,25OHVit D-induced upregulation of these and other genes in macrophages directly enhances killing of MTb and pseudomonas ^51^ among other organisms. 1,25OH-VitD also upregulates TLR and CD14 gene expression which together enhance detection of danger signals by the immune system. These mechanisms in part explain how keratinocytes ^52-53^ recognize invading pathogens during skin infections and support the concept that 25OH-Vit D deficiency could underlie enhanced susceptibility to bacterial infections in dialysis patients.

T cells ^22-23,26,54-55^ and DCs ^21,49^ also express the VDR and are responsive to active 1,25OH-VitD. 1,25OH-Vit D inhibits DC differentiation and maturation ^25^, lowering production of IL-12 and TNFa^24^ and limiting priming of Th1 immunity ^24^, directly prevents IFNg production by T cells ^56^, promotes Th2 immunity ^57-58^ and enhances regulatory T cell induction.^59^ Consistent with an immunomodulatory effect, 25OH-Vit D deficiency is associated with an elevated risk of inflammatory bowel disease ^60-62^, lupus ^63^, and Behcet’s disease ^64^ among other autoimmune disorders.^65^ Chronic kidney allograft injury is also more prevalent in Vit D-deficient patients.^66-68^

Some, but not all, studies suggest that therapy with active 1,25 Vit D may partially correct immune defects.^69-71^ We postulate that the effects of Vit D on the immune system require local (immune cell) conversion of 25OH-Vit to 1,25OH-Vit D, a process dependent upon adequate circulating levels of 25OH-Vit D. Thus, regardless of administered 1,25OH-VitD, deficiency of 25OH-Vit D will result in enhanced production of proinflammatory cytokines (IL12, TNFa), augmented Th1 immunity, upregulation of MHC, costimulatory molecules and adhesion molecules on DCs and defective intracellular killing of bacteria due to an inability to produce anti-microbial peptides. Our preliminary data demonstrate that Th1 alloimmunity is stronger in 25OH-VitD deficient dialysis patients, supporting this hypothesis and the proposed work.

STUDY AIMS

Primary Aim:

1.) Determine the effect of oral 25OH-Vit D treatment on change in 25OH-Vit D levels in Vit D deficient dialysis patients

Secondary Aims:

1.) Determine the effect of oral 25OH-Vit D treatment on change in PRT readout in Vit D deficient dialysis patients

2.) Determine the effect of oral 25OH-Vit D treatment on change in cytokine production by stimulated PBMCs in Vit D deficient dialysis patients

3.) Determine the effect of oral 25OH-Vit D treatment on change in PBMC profile in Vit D deficient dialysis patients

4.) Determine the effect of oral 25OH-Vit D treatment on response to influenza vaccination in Vit D deficient dialysis patients (substudy)

HYPOTHESES

1. Administration of oral 25OH-Vit D in 25OH-Vit D deficient dialysis patients results in normalization of 25OH-Vit D levels (Primary Aim).
2. Repletion of 25OH-Vit D in 25OH-Vit D deficient dialysis patients results in an overall improvement in immune function by limiting adaptive immunity and enhancing innate immunity (Secondary Aims 1 and 2).
3. Repletion of 25OH-Vit D in 25OH-Vit D deficient dialysis patients results in a change in PBMC profile (Secondary Aim 3).
4. Repletion of 25OH-Vit D in 25OH-Vit D deficient dialysis patients results in improved response to influenza vaccination secondary to enhancement of innate immune responses (Secondary Aim 4).

Trial Timeline****

|  | Screening/Enrollment | Randomization* | 6 weeks (+/2 weeks) | 3  months (+/- 2 weeks) | 6  months (+/- 2 weeks) | 12 months (+/- 2 weeks) | 13 months (+/- 2 weeks) |
| --- | --- | --- | --- | --- | --- | --- | --- |
| Informed consent | X |  |  |  |  |  |  |
| Routine history and physical (including vital signs, medication list, dialysis prescription) | X | X | X | X | X | X | X |
| 25OH-Vit D level (8 cc blood draw)*** | X |  | X | X | X | X |  |
| 43 cc blood draw*** (4 green top tubes and 1 red top tube, each 8 cc, + 3cc for RNA/PAX gene tube) for PBMC profile, PRT testing, CRP, serum cytokines, alloantibodies |  | X |  |  | X | X |  |
| Adverse Events |  | X | X | X | X | X | X |
| Oral Vit D3 (cholecalciferol)** |  | 50,000 IU Qweek | | 10,000 IU weekly | | |  |

* Only those study subjects with 25OH-Vit D deficiency (< 25 ng/mL) will proceed with randomization.

** Only for those study subjects randomized to oral Vitamin D therapy: those study subjects with 25OH-Vit D levels > 35 ng/mL will transition to maintenance therapy with 10,000 IU weekly at 6 weeks, whereas those study subjects who remain Vit D insufficient (< 35 ng/mL) will remain on doses of 50,000 IU weekly until reassessment at 3 months.

***Blood draws do not involve a needlestick as they will be done on dialysis.

**** In addition to the above tests, study subjects will have blood drawn (27 mL: one 8 mL red top tube and two 8 mL green top tubes) before and 2 months after influenza vaccination for assays of cellular immunity and antibodies to the species should they choose to receive the influenza vaccination during the season (offered as standard of care); or 2 months apart during influenza season should they refuse the vaccine.

**STUDY DESIGN AND PROCEDURES**

INCLUSION CRITERIA:

1. Age > 18 years
2. Chronic hemodialysis treatments for at least 2 consecutive months
3. 25OH-Vitamin D level < 25 ng/mL

EXCLUSION CRITERIA:

1. History of acute renal failure requiring dialysis with potential for renal recovery
2. History of HIV/AIDS
3. Inability to provide informed consent

NUMBER OF STUDY PARTICIPANTS:

We expect that 180 subjects will be screened in order to randomize approximately 140 subjects (approximately 84 subjects in the Vitamin D arm and 56 subjects in the control arm).

**Overview**

The primary objective of the study is to assess change in 25OH-Vit D levels in response to oral Vitamin D therapy (Vitamin D3: cholecalciferol) in deficient hemodialysis patients. 180 hemodialysis patients will be screened for Vitamin D deficiency, and those with Vitamin D levels < 25 ng/mL will be randomized to either 50,000 IU Vitamin D weekly or to no treatment (standard of care), and will be followed for the aforementioned outcomes.

**Enrollment/Screening**

Hemodialysis patients who meet the inclusion criteria for enrollment stated above will be identified at the dialysis unit by their physician during regularly scheduled visits. The potential subjects’ physicians will ask permission for the investigators to speak with the patient during their hemodialysis treatment for recruitment purposes. Those eligible patients who provide informed consent will be enrolled as study subjects. The enrollment period will last 1 year.

Consent from study subjects and controls will be obtained before any study-related procedures are performed. All potential subjects will be given consent documents written in the language s/he understands. Consent documents, including those translated into another language, will receive IRB approval prior to use. The investigator will explain the study and review the IRB-approved consent forms with the patient in detail. Each potential subject will have the opportunity to ask questions. Each potential subject will reiterate in his or her own words the purpose of the study, the risks, the benefits, and the alternatives to participating. Once the subject agrees to participate, s/he will sign and date the IRB consent forms. The investigator will also sign and date the consent forms. One copy of the consent forms will be given to the subject, another copy will be placed in his or her medical chart, and the original will be filed in the study’s research file.

Screening for 25OH-Vit D deficiency will be performed at the time of subject enrollment, after obtaining informed consent. Subjects with 25OH-Vit D levels > 25 ng/ml will be excluded from randomization and will be referred back to their dialysis physicians for further management and care.

**Randomization**

Randomization will be performed as the first activity of the baseline visit. Participants will be randomized to receive Vit D3 at a dose of 50,000 IU weekly or no treatment. Treatment allocation will be in a 2:1 ratio and will be stratified according to the subject’s 25OH-Vit D level: “moderately” Vit D deficient group of 25OH-Vit D levels 10-24 ng/ml and “severely” Vit D deficient group of 25OH-Vit D levels < 10 ng/ml. Randomized stratification will be used to minimize confounding by baseline Vitamin D level. Within each stratum, randomization will be done in 4-subject blocks. Treatment allocation will be open-label for safety considerations.

**Follow-up Visits**

Study visits will occur at baseline, 6 weeks, 3 months, 6 months, 12 months, and 13 months as described in the table above.

**Study Modality**

The Vitamin D3 will be supplied by Bio Tech Pharmacal, Inc. (www.Bio-Tech-Pharm.com) in the form of water-soluble capsules containing 50,000 IU of cholecalciferol (Vitamin D3) each. Inactive ingredients include microcrystalline cellulose, silica, and gelatin. The capsules will be delivered in safety-sealed bottles and will be stored in cool, dry conditions.

**Dosage and Administration**

Those participants randomized to oral Vitamin D will have a target Vitamin D level of > 35 ng/ml. The initial dose of Vit D3 will be 50,000 IU weekly for 6 weeks, at which point follow-up 25OH-Vit D levels will be measured. Those subjects who remain Vit D insufficient (25OH-Vit D < 35 ng/ml) will remain on 50,000 IU weekly for another 6 weeks, at which point Vit D levels will be repeated. Those subjects who achieve 25OH-Vit D levels > 35 ng/ml will be transitioned to 10,000 IU weekly.

**Study Procedures**

All participants (those receiving and those not receiving oral Vitamin D therapy) will have 8 cc of blood drawn for 25OH-Vit D level at enrollment. If they are found to be Vitamin D-deficient (25OH-Vit D < 25 ng/mL), they will have another 8 cc of blood taken at 6 weeks, 3 months, 6 months, and 12 months into the study for 25-OH Vit D levels. Furthermore, at randomization, 6 months, and 12 months, all study subjects will have 43 cc of blood drawn for PBMC profile, PRT testing, CRP, serum cytokines, and alloantibody testing (4 green top tubes, 1 red top tube, one PAX gene tube). All participants will be offered the influenza vaccination during flu season (standard of care), and will have blood drawn (27 mL: one 8 mL red top tube and two 8 mL green top tubes) before vaccination as well as two months after vaccination (or 2 months apart during flu season if the vaccination is declined) for assays of cellular immunity and antibodies to the species.

Serum calcium and intact PTH will be assessed at enrollment, 3 months, 6 months, and 12 months into the study (standard of care).

A history and physical examination will be performed on all study subjects at enrollment, randomization, 6 weeks, 3 months, 6 months, 12 month, and 13 months into the study. Patients will also be screened for adverse events at these time points.

**Study Completion or Withdrawal**

Each study subject will have completed the study 1 year after enrollment (at their final follow-up visit), and will be followed for an additional 30 days for adverse events. Study subjects are able to withdraw from the study at any time should they wish to withdraw. Withdrawal of study subjects may also occur at the discretion of the investigators.

PLANNED STATISTICAL ANALYSIS

The primary analysis will be done comparing those study subjects who became Vit D replete (Vit D > 35 ng/mL) to those study subjects who remained Vit D deficient/insufficient (Vit D < 35 ng/mL), regardless of treatment allocation. A secondary analysis will be performed on the basis of “intention to treat.” The primary outcome of change in 25OH-Vitamin D will be analyzed first using a student’s t-test. Potential confounders that will be examined include age, baseline 25OH-Vit D, time on hemodialysis, gender, and race. Those variables that differ significantly between treatment arms at an alpha of 0.01 and that are significantly related to the primary outcome at an alpha of 0.05 will be included in multivariable analysis. If any of the potential confounders meet the criteria for inclusion, the multivariable analysis will be the primary analysis. For the primary analysis, significance will be set at a two-tailed alpha of 0.05. Analysis of secondary outcomes will be performed in a similar fashion.

SAFETY EVALUATIONS

Assessment of Study Subjects

Subjects will be assessed for incidence and severity of adverse events during the study period. The following safety evaluations will be performed during the study to measure the safety and tolerability of oral 25OH-Vitamin D: serum calcium, intact PTH. Participants will be questioned at each study visit regarding any new health problems. Adverse events will be classified using the ICH classification system. All serious adverse events will be reported within 24 hours to the FDA and the Mount Sinai IRB. A quarterly review of safety outcomes/adverse events will occur in conjunction with an interim analysis of efficacy. In the event of a statistically significant increase in hypervitaminosis D (25OH-Vit D > 100 ng/ml) in either group, the study will be halted until appropriate safety measures can be taken. Furthermore, any trend towards statistical significance over time will also warrant temporary suspension of the study for further safety evaluations. Any temporary or permanent suspensions of the study will be reported to the Mount Sinai IRB and the FDA.

In order to further ensure patient safety, the 43 cc blood draw (see study procedures section above) will be deferred if the last available hemoglobin level (sent by the dialysis unit twice monthly as standard of care) is < 8.0 g/dL.

Safety Reporting Requirements and Responsibilities

**Adverse events**

Adverse events (AEs) will be recorded for the duration of the trial, regardless of whether or not the event(s) are considered related to trial medication. All AEs considered related to trial medication will be followed until resolution even if this occurs post-trial.

**Definitions of adverse events**

Adverse event (AE)

Any untoward medical occurrence in a clinical investigation patient administered a pharmaceutical product that does not necessarily have a causal relationship with this treatment. An adverse event (AE) can, therefore, be any unfavorable and unintended sign (including an abnormal finding), symptom, or disease temporally associated with the use of an investigational product, whether or not related to the investigational product.

Serious adverse event (SAE)

An adverse event occurring at any dose that results in any of the following outcomes:

-death

-a life-threatening adverse drug experience

-inpatient hospitalization or prolongation of existing hospitalization excluding those for study drug administration, transfusional support, disease staging/re-staging procedures, concomitant radiotherapy, thoracentesis/paracentesis, or placement of an indwelling catheter, unless associated with other serious events.

-persistent or significant disability/incapacity,

or

-congenital anomaly/birth defect.

Important medical events that may not result in death, be life-threatening, or require hospitalization may be considered serious adverse drug experiences when, based upon appropriate medical judgment, they may jeopardize the patient or subject and may require medical or surgical intervention to prevent one of the outcomes listed above.

Death, regardless of cause, which occurs within 30 days of the last dose of study drug or after 30 days and is a result of delayed toxicity due to administration of the study drug, should be reported as a serious adverse event.

**Reporting adverse events**

Adverse events

All adverse events will be recorded for the duration of a patient’s participation in the trial. All adverse events (including any laboratory abnormalities which require intervention), regardless of causal relationship, are to be reported to the institutional IRB. Pre-existing conditions at baseline will be recorded. If a pre-existing condition does not change, it does not have to be reported on subsequent cycles.

Serious adverse events

Adverse events classified as serious require expeditious handling and reporting to comply with regulatory requirements.

**Unlisted (Unexpected) Adverse Event**

An adverse event that is not mentioned in the Investigator's Brochure or package insert or the specificity or severity of which is not consistent with the Investigator's brochure or package insert

**Associated With the Use of the Drug**

An adverse event is considered associated with the use of the drug if the attribution is possible,

probable, or very likely by the definitions for an AE or SAE.

**Not related**

An adverse event which is not related to the use of the drug.

**Doubtful**

An adverse event for which an alternative explanation is more likely, e.g., concomitant drug(s),

concomitant disease(s), or the relationship in time suggests that a causal relationship is unlikely.

**Possible**

An adverse event that might be due to the use of the drug. An alternative explanation, e.g.,

concomitant drug(s), concomitant disease(s), is inconclusive. The relationship in time is

reasonable; therefore, the causal relationship cannot be excluded.

**Probable**

An adverse event which might be due to the use of the drug. The relationship in time is

suggestive (e.g., confirmed by de-challenge). An alternative explanation is less likely, e.g.,

concomitant drug(s), concomitant disease(s).

**Very likely**

An adverse event that is listed as a possible adverse reaction and cannot be reasonably explained

by an alternative explanation, e.g., concomitant drug(s), concomitant disease(s). The relationship

in time is very suggestive (e.g., it is confirmed by de-challenge and re-challenge).

Serious Adverse Event (SAE) Reporting

All serious adverse events will be reported to the appropriate regulatory agencies (FDA, study medication manufacturer) and the institution’s IRB.

**Study Drug Handling and Accountability**

The PI will be responsible for the handling and security of the study medication. The medication will be dispensed directly to patients at the hemodialysis unit by study staff.

Data Storage

Data will be stored on a secure computer within the Mount Sinai network in a database that will be password protected. Data will be identified by a unique research code. The link between the code and the identifying information will be stored in a locked file cabinet. Only the PI and co-investigators will have access to the link. The computer itself will be maintained in a locked fixed location in 21-32 of the Annenberg Building of Mount Sinai Hospital. Blood samples obtained for this study will not be stored for future research.

Ethical Obligations/Regulatory Guidelines

The study will not begin until IRB approval is received. All amendments to the study protocol will be approved by the IRB prior to their institution.

Study Monitoring

An independent study monitor has been appointed for this randomized controlled trial. This independent study monitor will be responsible for a quarterly review of safety outcomes/adverse events.

Data collection will be performed by qualified study team members. Data collected by these team members will be reviewed by the PI biweekly to assess accuracy and completeness. Missing data will be salvaged through an electronic medical record, when applicable.

Study Completion Procedures

The active study period will end after the last follow-up visit (1 year time-point) for the last patient randomized, with an additional 30-day follow-up period for adverse events. A review of the data will then take place followed by statistical analysis and manuscript preparation. This period of data analysis and manuscript preparation will continue for six months after the end of the study period.

REFERENCES

1. Griveas, I.*, et al.* Comparative analysis of immunophenotypic abnormalities in cellular immunity of uremic patients undergoing either hemodialysis or continuous ambulatory peritoneal dialysis. *Ren Fail* **27**, 279-282 (2005).

2. Griveas, I.*, et al.* Cellular immunity and levels of parathyroid hormone in uremic patients receiving hemodialysis. *Ren Fail* **27**, 275-278 (2005).

3. Hauser, A.B.*, et al.* Characteristics and causes of immune dysfunction related to uremia and dialysis. *Perit Dial Int* **28 Suppl 3**, S183-187 (2008).

4. Kuroki, Y.*, et al.* A study of innate immunity in patients with end-stage renal disease: special reference to toll-like receptor-2 and -4 expression in peripheral blood monocytes of hemodialysis patients. *Int J Mol Med* **19**, 783-790 (2007).

5. Lim, W.H., Kireta, S., Leedham, E., Russ, G.R. & Coates, P.T. Uremia impairs monocyte and monocyte-derived dendritic cell function in hemodialysis patients. *Kidney Int* **72**, 1138-1148 (2007).

6. Lonnemann, G. Impaired NK cell function in ESRD patients. *Blood Purif* **26**, 315-316 (2008).

7. Vacher-Coponat, H.*, et al.* Natural killer cell alterations correlate with loss of renal function and dialysis duration in uraemic patients. *Nephrol Dial Transplant* **23**, 1406-1414 (2008).

8. Pahl, M.V.*, et al.* Effect of end-stage renal disease on B-lymphocyte subpopulations, IL-7, BAFF and BAFF receptor expression. *Nephrol Dial Transplant* (2009).

9. Raska, K., Jr.*, et al.* T cell subsets and cellular immunity in end-stage renal disease. *Am J Med* **75**, 734-740 (1983).

10. Raskova, J.*, et al.* B-cell activation and immunoregulation in end-stage renal disease patients receiving hemodialysis. *Arch Intern Med* **147**, 89-93 (1987).

11. Song, J.Y.*, et al.* Active influenza immunization in hemodialysis patients: comparison between single-dose and booster vaccination. *Am J Nephrol* **26**, 206-211 (2006).

12. van Dijk, P.C.*, et al.* Renal replacement therapy in Europe: the results of a collaborative effort by the ERA-EDTA registry and six national or regional registries. *Nephrol Dial Transplant* **16**, 1120-1129 (2001).

13. Adams, J.S. & Hewison, M. Unexpected actions of vitamin D: new perspectives on the regulation of innate and adaptive immunity. *Nat Clin Pract Endocrinol Metab* **4**, 80-90 (2008).

14. Bikle, D. Nonclassic actions of vitamin D. *J Clin Endocrinol Metab* **94**, 26-34 (2009).

15. Bikle, D.D. Vitamin D and the immune system: role in protection against bacterial infection. *Curr Opin Nephrol Hypertens* **17**, 348-352 (2008).

16. Cantorna, M.T., Yu, S. & Bruce, D. The paradoxical effects of vitamin D on type 1 mediated immunity. *Mol Aspects Med* **29**, 369-375 (2008).

17. Chun, R.F., Adams, J.S. & Hewison, M. Back to the future: a new look at 'old' vitamin D. *J Endocrinol* **198**, 261-269 (2008).

18. van Etten, E. & Mathieu, C. Immunoregulation by 1,25-dihydroxyvitamin D3: basic concepts. *J Steroid Biochem Mol Biol* **97**, 93-101 (2005).

19. White, J.H. Vitamin D signaling, infectious diseases, and regulation of innate immunity. *Infect Immun* **76**, 3837-3843 (2008).

20. Jean, G.*, et al.* Daily oral 25-hydroxycholecalciferol supplementation for vitamin D deficiency in haemodialysis patients: effects on mineral metabolism and bone markers. *Nephrol Dial Transplant* **23**, 3670-3676 (2008).

21. Hewison, M.*, et al.* Differential regulation of vitamin D receptor and its ligand in human monocyte-derived dendritic cells. *J Immunol* **170**, 5382-5390 (2003).

22. Lemire, J.M.*, et al.* 1,25-Dihydroxyvitamin D3 suppresses human T helper/inducer lymphocyte activity in vitro. *J Immunol* **134**, 3032-3035 (1985).

23. Lemire, J.M., Adams, J.S., Sakai, R. & Jordan, S.C. 1 alpha,25-dihydroxyvitamin D3 suppresses proliferation and immunoglobulin production by normal human peripheral blood mononuclear cells. *J Clin Invest* **74**, 657-661 (1984).

24. Penna, G.*, et al.* 1,25-Dihydroxyvitamin D3 selectively modulates tolerogenic properties in myeloid but not plasmacytoid dendritic cells. *J Immunol* **178**, 145-153 (2007).

25. Piemonti, L.*, et al.* Vitamin D3 affects differentiation, maturation, and function of human monocyte-derived dendritic cells. *J Immunol* **164**, 4443-4451 (2000).

26. Provvedini, D.M., Tsoukas, C.D., Deftos, L.J. & Manolagas, S.C. 1,25-dihydroxyvitamin D3 receptors in human leukocytes. *Science* **221**, 1181-1183 (1983).

27. Provvedini, D.M., Tsoukas, C.D., Deftos, L.J. & Manolagas, S.C. 1 alpha,25-Dihydroxyvitamin D3-binding macromolecules in human B lymphocytes: effects on immunoglobulin production. *J Immunol* **136**, 2734-2740 (1986).

28. Rigby, W.F., Stacy, T. & Fanger, M.W. Inhibition of T lymphocyte mitogenesis by 1,25-dihydroxyvitamin D3 (calcitriol). *J Clin Invest* **74**, 1451-1455 (1984).

29. Zarrabeitia, M.T., Riancho, J.A., de Francisco, A.L. & Gonzalez-Macias, J. Effect of physiological concentrations of calcitriol on lymphocyte proliferation in normal subjects and in patients with renal failure. *Nephron* **55**, 110-113 (1990).

30. Sarnak, M.J. & Jaber, B.L. Mortality caused by sepsis in patients with end-stage renal disease compared with the general population. *Kidney Int* **58**, 1758-1764 (2000).

31. Cavdar, C.*, et al.* The comparison of antibody response to influenza vaccination in continuous ambulatory peritoneal dialysis, hemodialysis and renal transplantation patients. *Scand J Urol Nephrol* **37**, 71-76 (2003).

32. Girndt, M., Pietsch, M. & Kohler, H. Tetanus immunization and its association to hepatitis B vaccination in patients with chronic renal failure. *Am J Kidney Dis* **26**, 454-460 (1995).

33. Kreft, B., Klouche, M., Kreft, R., Kirchner, H. & Sack, K. Low efficiency of active immunization against diphtheria in chronic hemodialysis patients. *Kidney Int* **52**, 212-216 (1997).

34. Chia, S., Karim, M., Elwood, R.K. & FitzGerald, J.M. Risk of tuberculosis in dialysis patients: a population-based study. *Int J Tuberc Lung Dis* **2**, 989-991 (1998).

35. Chou, K.J.*, et al.* Tuberculosis in maintenance dialysis patients. *Nephron* **88**, 138-143 (2001).

36. Simon, T.A., Paul, S., Wartenberg, D. & Tokars, J.I. Tuberculosis in hemodialysis patients in New Jersey: a statewide study. *Infect Control Hosp Epidemiol* **20**, 607-609 (1999).

37. Liu, P.T.*, et al.* Toll-like receptor triggering of a vitamin D-mediated human antimicrobial response. *Science* **311**, 1770-1773 (2006).

38. Ando, M., Shibuya, A., Tsuchiya, K., Akiba, T. & Nitta, K. Reduced expression of Toll-like receptor 4 contributes to impaired cytokine response of monocytes in uremic patients. *Kidney Int* **70**, 358-362 (2006).

39. Meuer, S.C., Hauer, M., Kurz, P., Meyer zum Buschenfelde, K.H. & Kohler, H. Selective blockade of the antigen-receptor-mediated pathway of T cell activation in patients with impaired primary immune responses. *J Clin Invest* **80**, 743-749 (1987).

40. Stachowski, J., Pollok, M., Burrichter, H., Spithaler, C. & Baldamus, C.A. Signalling via the TCR/CD3 antigen receptor complex in uremia is limited by the receptors number. *Nephron* **64**, 369-375 (1993).

41. Stachowski, J.*, et al.* Defective antigen presentation by monocytes in ESRD patients not responding to hepatitis B vaccination: impaired HBsAg internalization and expression of ICAM-1 and HLA-DR/Ia molecules. *Mediators Inflamm* **4**, 49-54 (1995).

42. Stachowski, J., Pollok, M., Barth, C., Maciejewski, J. & Baldamus, C.A. Non-responsiveness to hepatitis B vaccination in haemodialysis patients: association with impaired TCR/CD3 antigen receptor expression regulating co-stimulatory processes in antigen presentation and recognition. *Nephrol Dial Transplant* **9**, 144-152 (1994).

43. Stachowski, J., Pollok, M., Burrichter, H. & Baldamus, C.A. Immunodeficiency in ESRD-patients is linked to altered IL-2 receptor density on T cell subsets. *J Clin Lab Immunol* **34**, 171-177 (1991).

44. Sester, U.*, et al.* T-cell activation follows Th1 rather than Th2 pattern in haemodialysis patients. *Nephrol Dial Transplant* **15**, 1217-1223 (2000).

45. Descamps-Latscha, B.*, et al.* Balance between IL-1 beta, TNF-alpha, and their specific inhibitors in chronic renal failure and maintenance dialysis. Relationships with activation markers of T cells, B cells, and monocytes. *J Immunol* **154**, 882-892 (1995).

46. Eleftheriadis, T., Antoniadi, G., Liakopoulos, V., Kartsios, C. & Stefanidis, I. Disturbances of acquired immunity in hemodialysis patients. *Semin Dial* **20**, 440-451 (2007).

47. Wolf, M.*, et al.* Vitamin D levels and early mortality among incident hemodialysis patients. *Kidney Int* **72**, 1004-1013 (2007).

48. Chen, S., Sims, G.P., Chen, X.X., Gu, Y.Y. & Lipsky, P.E. Modulatory effects of 1,25-dihydroxyvitamin D3 on human B cell differentiation. *J Immunol* **179**, 1634-1647 (2007).

49. Griffin, M.D. & Kumar, R. Effects of 1alpha,25(OH)2D3 and its analogs on dendritic cell function. *J Cell Biochem* **88**, 323-326 (2003).

50. Manolagas, S.C., Provvedini, D.M. & Tsoukas, C.D. Interactions of 1,25-dihydroxyvitamin D3 and the immune system. *Mol Cell Endocrinol* **43**, 113-122 (1985).

51. Wang, T.T.*, et al.* Cutting edge: 1,25-dihydroxyvitamin D3 is a direct inducer of antimicrobial peptide gene expression. *J Immunol* **173**, 2909-2912 (2004).

52. Schauber, J.*, et al.* Histone acetylation in keratinocytes enables control of the expression of cathelicidin and CD14 by 1,25-dihydroxyvitamin D3. *J Invest Dermatol* **128**, 816-824 (2008).

53. Schauber, J.*, et al.* Injury enhances TLR2 function and antimicrobial peptide expression through a vitamin D-dependent mechanism. *J Clin Invest* **117**, 803-811 (2007).

54. Bhalla, A.K., Amento, E.P. & Krane, S.M. Differential effects of 1,25-dihydroxyvitamin D3 on human lymphocytes and monocyte/macrophages: inhibition of interleukin-2 and augmentation of interleukin-1 production. *Cell Immunol* **98**, 311-322 (1986).

55. Bhalla, A.K., Amento, E.P., Serog, B. & Glimcher, L.H. 1,25-Dihydroxyvitamin D3 inhibits antigen-induced T cell activation. *J Immunol* **133**, 1748-1754 (1984).

56. Helming, L.*, et al.* 1alpha,25-Dihydroxyvitamin D3 is a potent suppressor of interferon gamma-mediated macrophage activation. *Blood* **106**, 4351-4358 (2005).

57. Lemire, J.M., Archer, D.C., Beck, L. & Spiegelberg, H.L. Immunosuppressive actions of 1,25-dihydroxyvitamin D3: preferential inhibition of Th1 functions. *J Nutr* **125**, 1704S-1708S (1995).

58. Boonstra, A.*, et al.* 1alpha,25-Dihydroxyvitamin d3 has a direct effect on naive CD4(+) T cells to enhance the development of Th2 cells. *J Immunol* **167**, 4974-4980 (2001).

59. Adorini, L., Penna, G., Giarratana, N. & Uskokovic, M. Tolerogenic dendritic cells induced by vitamin D receptor ligands enhance regulatory T cells inhibiting allograft rejection and autoimmune diseases. *J Cell Biochem* **88**, 227-233 (2003).

60. Podolsky, D.K. Inflammatory bowel disease (2). *N Engl J Med* **325**, 1008-1016 (1991).

61. Podolsky, D.K. Inflammatory bowel disease (1). *N Engl J Med* **325**, 928-937 (1991).

62. Sonnenberg, A., McCarty, D.J. & Jacobsen, S.J. Geographic variation of inflammatory bowel disease within the United States. *Gastroenterology* **100**, 143-149 (1991).

63. Kamen, D.L.*, et al.* Vitamin D deficiency in systemic lupus erythematosus. *Autoimmun Rev* **5**, 114-117 (2006).

64. Do, J.E., Kwon, S.Y., Park, S. & Lee, E.S. Effects of vitamin D on expression of Toll-like receptors of monocytes from patients with Behcet's disease. *Rheumatology (Oxford)* **47**, 840-848 (2008).

65. Munger, K.L., Levin, L.I., Hollis, B.W., Howard, N.S. & Ascherio, A. Serum 25-hydroxyvitamin D levels and risk of multiple sclerosis. *JAMA* **296**, 2832-2838 (2006).

66. Becker, B.N.*, et al.* Vitamin D as immunomodulatory therapy for kidney transplantation. *Transplantation* **74**, 1204-1206 (2002).

67. Hullett, D.A.*, et al.* Prevention of chronic allograft nephropathy with vitamin D. *Transpl Int* **18**, 1175-1186 (2005).

68. O'Herrin, J.K., Hullett, D.A., Heisey, D.M., Sollinger, H.W. & Becker, B.N. A retrospective evaluation of 1,25-dihydroxyvitamin D(3) and its potential effects on renal allograft function. *Am J Nephrol* **22**, 515-520 (2002).

69. Adams, J.S.*, et al.* Vitamin d-directed rheostatic regulation of monocyte antibacterial responses. *J Immunol* **182**, 4289-4295 (2009).

70. Gregori, S., Giarratana, N., Smiroldo, S., Uskokovic, M. & Adorini, L. A 1alpha,25-dihydroxyvitamin D(3) analog enhances regulatory T-cells and arrests autoimmune diabetes in NOD mice. *Diabetes* **51**, 1367-1374 (2002).

71. Zhu, Y., Mahon, B.D., Froicu, M. & Cantorna, M.T. Calcium and 1 alpha,25-dihydroxyvitamin D3 target the TNF-alpha pathway to suppress experimental inflammatory bowel disease. *Eur J Immunol* **35**, 217-224 (2005).
