# Supplementary material for: Effect of Cholecalciferol Supplementation on Inflammation and Cellular Alloimmunity in Hemodialysis Patients: Data from a Randomized Controlled Pilot Trial
Source: PLoS One. 2014 Oct 8;9(10):e109998. doi: 10.1371/journal.pone.0109998 (PMC4190314; doi:10.1371/journal.pone.0109998)
Supplement: Sample Case Report Form S1 — Sample case report form from the trial used for data collection. (DOCX) [file pone.0109998.s006.docx]

**GCO #: 09-2275**

***Patient Name: _____________________________ Dialysis Shift*** *(circle)****: 94st TCC VA***

**Patient ID: ­­________________________________** MWF1 MWF2 MWF 3 MWF 4

**Date of Enrollment: ­­­­­­­­­________________________** TTS1 TTS2 TTS3 TTS4

**Age: _____________ Gender** (circle one)**:** M F **Hispanic** (circle)**:**  Y N

**Race** (circle one)**:** White Black/African-American Asian

American Indian/Alaska Native Native Hawaiian or Other Pacific Islander Other/Mixed

**Time on dialysis** (in years + months)**: __________ Etiology of ESRD:_____________**

**History of Kidney Transplant** (circle)**:** Y N **Number** **of Tx____ History of Transfusion** (circle)**:** Y N

**History of Pregnancy** (including abortions/miscarriages; circle)**:** Y N **Number** of pregnancies_____

**Diabetes** (circle)**:** Y N **Coronary Artery Disease** (circle)**:** Y N

**Peripheral Vascular Disease** (circle)**:** Y N **Hepatitis B** (circle)**:** Y N **Hepatitis C** (circle)**:** Y N

**Current Dialysis Access** (circle one)**:** AVF AVG catheter

**During PAST 12 months have you had: a) Dialysis Access Infection?** (circle one)**:**Y N

**b) Dialysis Access Thrombosis?** Y N c) **Number of IR declotting procedures? __________**

**d) Infections & how many bouts (choose all): bacteremia___, pneumonia___, cellulitis___, other _________**

**Number of Hospitalizations in the last six months: ­­­­­­­­­_______ Last 12 months: ______**

**Smoking status: [ ] Current smoker [ ] Remote [ ] Never Number of pack-years:____________**

**Medications: 1,25 Vit D Analog and Dose: Zemplar___________or Hectorol_________or Calcitriol________**

**EPO Analog and Dose: Darbepoetin (Aranesp) _________________or EPO________________**

**Phosphorus Binders & Dose: Phoslo _________ Renvela_________ Renagel_________ Fosrenal_______**

**Calcimimetic and Dose: Sensipar_________________________**

**ACE/ARB & Dose: Lisinopril_____or Enalapril_______ Cozaar______ or Diovan______ or other________**

**Statin & Dose: Zocor_______ Lova/prava/simva-statin _______ Other__________________**

**Other BP meds/Doses: Metoprolol______ Toprol______Nifedipine______Clonidine_____Hydralazine______**

**Aspirin________________ Plavix_______________ Coumadin_____________________**

**Oral vitamins and Dose: Nephrovite(renal vitamin)_________ or Multivitamin_________ or other_______**
